# Attachment anxiety benefits from security priming: Evidence from working memory performance

**DOI:** 10.1371/journal.pone.0193645

**Published:** 2018-03-09

**Authors:** Ahu Gokce, Mehmet Harma

**Affiliations:** Department of Psychology, Kadir Has University, Istanbul, Turkey; Technion Israel Institute of Technology, ISRAEL

## Abstract

The present study investigates the relationship between the attachment dimensions (anxious vs. avoidance) and the cognitive performance of individuals, specifically whether the attachment dimensions would predict the working memory (WM) performance. In the n-back task, reflecting the WM capacity, both attachment related and non-attachment related words were used. Participants were randomly assigned into two groups that received either the secure or the neutral subliminal priming. In the secure priming condition, the aim was to induce sense of security by presenting secure attachment words prior to the n-back task performance. In neutral priming condition, neutral words that did not elicit sense of security were presented. Structural equation modeling revealed divergent patterns for attachment anxiety and avoidance dimensions under the different priming conditions. In neutral priming condition, WM performance declined in terms of capacity in the n-back task for individuals who rated higher levels of attachment anxiety. However in the secure priming condition, WM performance was boosted in the n-back task for individuals who rated higher levels of attachment anxiety. In other words, the subliminal priming of the security led to increased WM capacity of individuals who rated higher levels of attachment anxiety. This effect, however, was not observed for higher levels of attachment avoidance. Results are discussed along the lines of hyperactivation and deactivation strategies of the attachment system.

## Introduction

A number of psychological traditions have focused on several stages of reciprocal relationship and their consequences from different perspectives. John Bowlby (1969) introduced his groundbreaking comprehensive theory, the attachment theory, to explain the nature of interpersonal interactions by synthesizing a wide-range of research traditions, including psychoanalysis, control system theory, ethology, and cognitive psychology. Drawing from a combination of these rich intellectual traditions, he defined the attachment concept as a biologically-based behavioral system which has survival value across life courses. Bowlby [1] proposed that human beings are equipped with behavioral systems each having its own functions and set-goals and he devoted the most attention to the attachment behavioral system and its goals. These goals are thought to protect a person from external dangers by ensuring proximity to the protective and/or caring others that are called the attachment figures.

In addition, he asserted that the attachment behavioral system is modified and re-organized through countless interactions with caregivers. In 1970s, “secure base” concept was introduced [2] and based on the observational studies, a number of attachment patterns were defined among infants: secure, avoidant and anxious attachment. Attachment theory was also extended to understand the adult romantic relationship by Hazan and Shaver’s [3] pioneering study after which research on adult attachment has been proliferated, especially in the domain of individual differences in the attachment orientations. As a result of exclusive research interest in individual differences, Bowlby’s [1] propositions about normative functioning of the attachment system corresponding to the universal aspect of the behavioral system has remained relatively unexplained.

Drawing on these previous works on both normative processes and individual differences, Shaver and Mikulincer [4] identified strategies regarding the activation of the attachment behavioral system: the primary and secondary strategies. The primary strategies refer to the perceived availability and responsiveness of the attachment figure and they are thought to be associated with the development of security-based strategies. The present study’s focus is on the activation of the secondary strategies which are grouped into two, namely the hyperactivation and deactivation strategies. The hyperactivation strategy involves exaggerating attempts to seek proximity with the attachment figure, which is typically attributed to anxious individuals (i.e., those who score high on anxiety and low on avoidance). This strategy is learned early in life to make certain that an inconsistent, distracted, or unreliable caregiver pays attention, and offers protection and support [2]. Another secondary strategy, deactivation strategy, involves giving up the proximity-seeking attempts and preferring excessive self-reliance to deal with distress, which is identified with avoidant individuals (i.e., those who score low on anxiety and high on avoidance). This strategy is learned in the context of a caregiver who provides better protection when one does not complain and does not insist on close contact [2].

### The associations between secondary strategies and the cognitive performance

Bowlby [5] and Ainsworth ([2] have extensively focused on the attachment behavioral system, viewed as the basic component of the emotional bonding between infants and their primary caregivers. According to Bowlby [6], the attachment and the other behavioural systems are closely related. When the core attachment needs such as separation distress reduction and proximity seeking are met, the remaining resources can be allocated to the other behavioural systems such as exploration. The function of the attachment behavioural systems is thought to be survival.

Consistent with these arguments, in a self-report study, Mikulincer [7] showed significant associations between attachment style and curiosity, which could be one of the indicators of the exploration of the behavioral system. Specifically, he found that attachment avoidance was associated with less curiosity, compared to those with high attachment anxiety. Consistently, previous work has also shown that individuals who reported higher levels of attachment avoidance could divert their attention away from attachment related stimuli [8,9], have more errors [10] and greater difficulty in encoding and recalling process for such kind of information [11, 12, 13]. These previous works indicate the functioning of a preemptive or cognitive-control strategy [14]. Preemptive defensive strategies refer to the avoidance of an unwanted memory or feeling that may potentially evoke frustration. These strategies are used to limit the amount of information available for encoding [12]. However, it has remained unclear whether this strategic use of cognitive source is only limited to the attachment-related content or not. Thus, we examined the association between attachment avoidance and cognitive performance using a cognitive task with and without attachment related stimuli.

The relationship between attachment orientations and cognitive performance has been addressed in various studies albeit focusing on different contents. For instance, in a longitudinal study [15] children with secure and insecure attachment styles were tested in reasoning tasks and results showed that securely attached children performed better in the reasoning task in adulthood compared to insecurely attached children.

Additionally, attachment anxiety was associated with different pattern when the attachment system is activated in various cognitive tasks. Specifically, attachment anxiety (characterized by hyperactivation strategy) predicted greater difficulty in disengaging from attachment-related stimuli and increased false alarm rate in a signal detection task [10]. Contrary to the individuals with higher levels of avoidance, those with high attachment anxiety are faster in processing attachment related cues when both stress and neutral primes are presented [16]. Similarly, Dewitte, Koster, De Houwer, and Buysse [17] showed that attachment anxiety is also related to attentional bias toward attachment figures under both threat and positive attachment conditions. Taken together, these results indicate that anxiously attached people exhibit chronic hyperactivation of the attachment system, which is easily triggered even when there is no apparent signal of threat.

Attachment avoidance, on the other hand, has been found to be associated with shifting attention away from stimuli depicting or evoking attachment-related cues such as mother or ex-partner pictures [8, 9]. When cognitive load is imposed, the ability of avoidant people’s disregarding such information disappears [18, 19], indicating that avoidant people may have presumably practiced defensive attentional control (i.e., preemptive strategies).

Considering preemptive strategies of avoidant people and increased accessibility to attachment-related cues of anxious people, there should be some possible strategies for anxious people to perform better in a cognitive task, if provided with the sense of security. Thus, we examined if security priming would facilitate the cognitive performance of individuals who rated higher levels of attachment anxiety.

### Priming sense of security and its consequences

Previous studies have consistently shown that priming security-related cognitions could be seen as equivalent to exposure to an attachment figure and this process is thought to create some effects that could mirror dispositional attachment security characteristics [19]. On the one hand, supraliminal priming techniques involve asking participants to imagine and explain their memories of feeling supported by an attachment figure [20]. On the other hand, subliminal priming techniques refer to the process in which participants are exposed to words or pictures related to secure attachment (see [21] for a review). These priming procedures have been administered in various studies to exert attachment figure effects through spreading activation [22]. Cumulative research yielded significant impacts of secure priming. For instance, security priming was associated with positive mood, relationship goals (e.g., positive expectations about their relationship), and more favorable self-views [20] greater felt security [23]; less hostile attitude toward outgroup members [21, 24]; and reduced prejudice toward outgroup members [25]. Besides, Gillath, Selcuk, and Shaver [26] showed that positive effects of repeated security priming could be sustained at least for a few days. In the current study, we used subliminal priming to investigate how priming may contribute to the cognitive performance. Specifically, we examined the role of attachment dimensions on working memory performance (manifested in n-back task) under different priming conditions.

### Cognition and working memory

As stated in the previous sections, the attachment behavioral system is closely related with the activated cognition systems under various conditions [27]. Human cognitive system moderates the multiple environmental inputs that one can face in a given moment. These inputs could or could not necessarily be relevant to the current goal of the person. Cognitive processes such as maintaining present and future goals, switching between different tasks, filtering out irrelevant information, keeping new information for limited time in mind enable to perform the duties [28].. Working memory (WM), being one of these processes, refers to the maintenance and active manipulation of information in memory for temporary period [29]. The number of items one can hold in memory (WM capacity) and duration of keeping those items in memory are the two key features of WM. Both of these factors are limited, that is the number of items that can be hold are small in quantity (7±2 items [30] or 4 chunks [31] and the duration of keeping those items is only temporary. In a given WM task, participants are presented with information (e.g., set of items), asked to keep that information in memory and process to later retrieve and perform the task. The basic components to perform a WM task are storage, maintenance, and retrieval which can be modulated by factors such as task type, processing speed, cognitive strategies, cognitive load, and attentional mechanisms [32, 33, 34]. Applied to the present study, if the sense of security provides extra room for cognitive performance, it could be expected that the person may benefit from the security priming in working memory tasks.

There are various tasks to measure the WM capacity such as operation span task, complex span task, reading span, and comprehension task, n-back task, arithmetic and verbal tasks, dual task paradigms, recall and recognition tasks, spatial tasks, etc. Individual differences in the WM task performance in various domains such as updating information [35, 36, 37], intelligence [33], language abilities [38], and conflict monitoring [39] have been shown. The observed differences in these tasks reveal individual differences that is reflected on the behavioral performances and leading to a distinction between low vs. high WM capacity.

### The present study

In this study we aim to investigate the association between the attachment dimensions (i.e., avoidance and anxiety) and WM performance by using the n-back WM task [40]. Previous research has shed light on the relationship between cognitive performance and attachment orientation of individuals [7, 41] Across multiple studies investigating the information processing of individuals along the curiosity domain, Mikulincer [7] found positive correlation between attachment security and cognitive performance including searching for new information and being flexible. Related to the present study, Edelstein [41] investigated the processing of attachment related and non-related words (emotional and neutral) of individuals with avoidant attachment orientation using WM task. In the study WM capacity was measured by analyzing the number of correctly recalled words. The results revealed that high attachment avoidance led to decline in the WM capacity when the task was to recall attachment-related words compared to non-attachment related words.

In the present study, we specifically tested the WM capacity assessed by the n-back task. The dependent variable in an n-back task is the accuracy of the responses. In order to measure and interpret the accuracy results, signal detection theory (SDT) gives the most informative value named as the d’ value reflecting sensitivity in the response (see [42] for a discussion on the validity of the d’ measurement in n-back task albeit in a sample with psychopathology). Higher d’ values indicate that participants have higher sensitivity in detecting differences as reflected in accuracy, while lower d’ values would refer that participants have lower sensitivity in detecting differences between the stimuli. According to SDT, in a task that requires making a simple yes/no judgment, there are different response outcomes: hit, miss, correct rejection, and false alarm. Applied to the n-back task, if the participant responds correctly based on the judgment of whether the item is same as *n* trials before, this would result in hit (responding to the presence of match) or correct rejection (responding to the absence of match). However if the participant responds incorrectly, this would result in miss (missing the presence of the match) or false alarm (thinking there was a match although there was not) [43] The high reliability of the SDT, its common use in various paradigms, its ability to measure the sensitivity in n-back WM task and reflecting individual differences precisely makes it a suitable method to use in the current study. The changes in the d’ values will enable to quantify the task performance under increasing WM load conditions predicted by the attachment dimensions. In addition to the d’ measurement, reaction time (RT) performance in the n-back task, reflecting the processing speed, was assessed as well.

Overall, the aim of the present study is to investigate the differences in working memory performance in an n-back task predicted by the avoidant and anxious attachment dimensions under different priming conditions. Previous studies [26, 44, 7] have focused on the relationship between cognition and attachment dimensions however to the best of our knowledge, there are no findings reported on the measurement of WM performance by the n-back task and that used attachment related and non-attachment related words as stimuli. More specifically, we investigated how the security priming would influence the association between attachment dimensions (i.e., anxiety and avoidance) and WM performance by using different word contents (i.e., attachment and non-attachment related words) under neutral and secure priming conditions.

The hypotheses of the present study are as follows:

1. n-back task performance will differ under different priming conditions. In the secure priming condition, participants scoring high on attachment anxiety will perform better (faster RTs and higher d’ values) compared to neutral priming condition for both word types.
2. No significant association between attachment avoidance and n-back task performance in the different priming conditions was hypothesized due to the use of hyperactivation strategies.
3. In accordance with the findings of Edelstein [41], we expected higher levels of attachment avoidance to predict poorer n-back task performance for attachment-related words.

## Method

### Participants

Eighty-four participants participated in the study (female: 65, *M*_*age*_ = 22 years, *SD* = 2 years). The mean value for anxiety dimension was 3.27 (SD = .92) and for avoidance was 2.57 (SD = .94). They were recruited from Kadir Has University. All participants had normal or corrected-to-normal vision. They were naïve as to the purpose of the experiment, and were debriefed afterwards about the study. Conducting this study was approved by the Ethics Committee of the Kadir Has University. Participants gave written consent prior to their participation and the anonymity of their recorded and stored response and self-report data were guaranteed. Participants in the sample enrolled in the psychology undergraduate program received course credits for their participation.

### Materials

Attachment dimensions were measured by the Turkish translation of ECR-R [45]. The scale assesses the adult attachment anxiety and avoidance dimensions [12]. The avoidance subscale (18 items; α = .90) measures the extent of an individual’s discomfort with closeness, dependence, and self- disclosure (e.g., ‘*I am nervous when my partner gets too close to me’*). The anxiety subscale (18 items; α = .86) refers to a strong need for closeness, fear of being abandoned, and rejection (e.g., ‘*I often worry that my partner doesn’t really love me*’). Participants indicated their level of agreement with each item on a 7-point Likert (disagree-agree) scale. The mean scores of each dimension were calculated and the higher score in each scale refers to higher levels of attachment related anxiety and avoidance.

Stimulus presentation and response recording were controlled by a HP ProDesk PC running with the Microsoft Windows 10 operating system. The experiment was built by using the PsychoPy Builder software (version 1.84.2) [46, 47]. Participants responded via the computer keyboard placed in front of them. The distance between the participant and the screen was approximately 60 cm and the screen resolution was set to 1920 x 1080 pixels. The experiment room was dimly lighted during testing. In one of the experiment rooms, 3 out of 4 PCs were used simultaneously during testing. Each desk and monitor was separated by a visual obstacle located next to each participant’s monitor to prevent sight of other participants and a curtain to prevent potential visual distraction in the room.

All the prime words and the n-back task words were presented in Turkish. The prime words were translated from the study of Mikulincer and Shaver [24] (neutral prime words: boat, table, picture, office; secure prime words: love, hugging, trust, closeness).

In an n-back task, people are presented with sequence of stimuli such as digits, letters, visuospatial locations, or words. The task is to respond whether the current presented item is the same as the item that was presented *n* trials before. The response is based on whether or not there is a match between the current trial item and the *n* times *back* presented item. The task measures the WM capacity and the number *n* is a factor that represents holding information for certain duration, manipulating and retrieving it to respond. For example, in an n-1 task participants should compare the current item to the item presented 1 trial before, in n-2 task they compare the current item to the one presented 2 trials before etc. As the number of trials going “back” increase, the task gets more difficult due to the limited capacity of WM and the accuracy performance starts to deteriorate. The *n* number varies between 0 and 3 (n-0, n-1, n-2, n-3); n-0 serves as control condition and ascending numbers 1–3 represent the increase in WM load. Using higher numbers (n-4, n-5 and so on) would make the task too difficult and impossible to perform as the capacity would be exceeded so researchers use maximum 3-back (i.e. n-3) condition. In each n-back task block (n-1, n-2, n-3), attachment related (eg. engagement, devotion, passion) or non-attachment related (eg. cheese, box, shoe) words were presented. The non-attachment related n-back task words were chosen from the database of Turkish Word Norms [48]. We chose the words that had comparable word length and that had the highest values on the word frequency dimension. The prime words were presented group-wise and the n-back task words were presented within all participants.

Before choosing the attachment related words, we generated a list with 80 words related to romantic relationship and asked four researchers to rate the relevance of these words from 1 (not related) to 7 (highly related). The inter-rater reliability of evaluators was .76. We chose the 50 words with the highest scores for attachment relatedness for the current study. In total, 50 attachment-related and 50 non-attachment related words were used.

### Procedure

**Fig 1:**
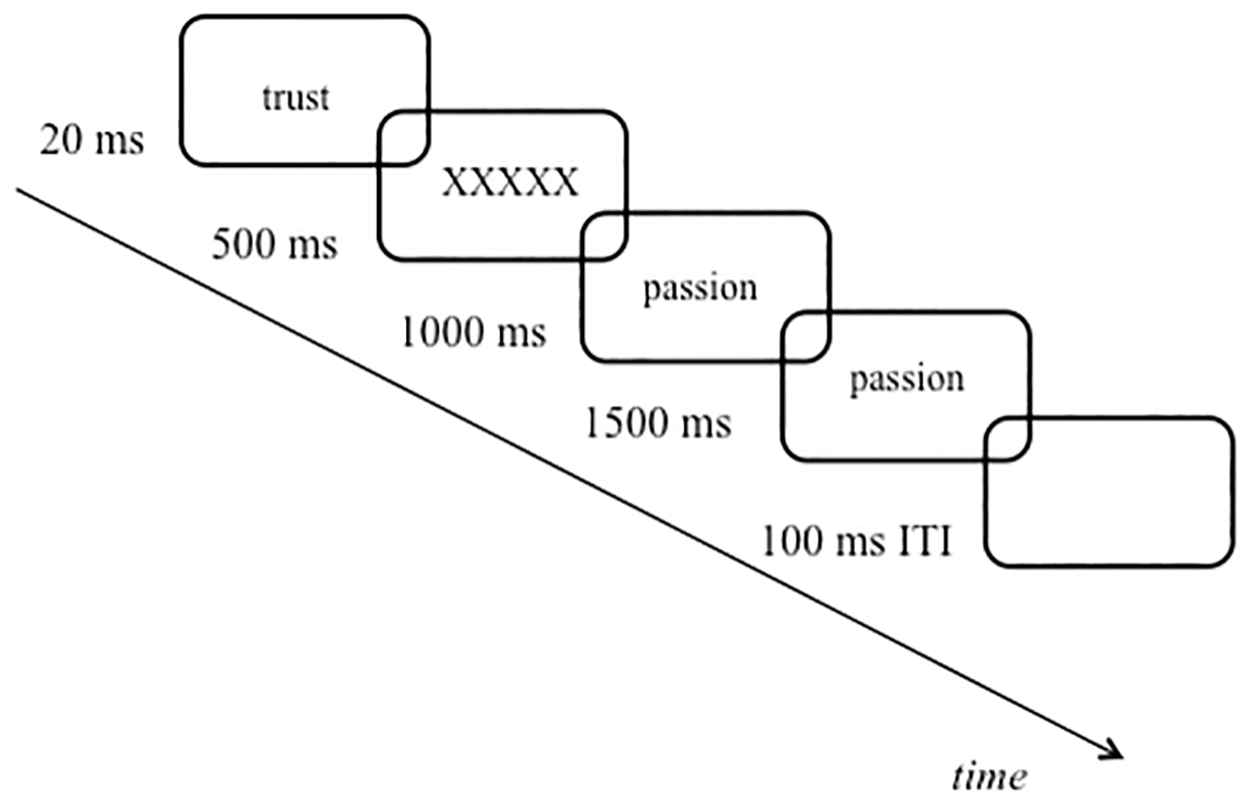
Task sequence. The depiction of the n-back task used in the present study. Here participants were subliminally primed with the secure attachment word (trust), followed by the presentation of the mask, they performed the n-back task with the attachment related words (passion). The task was to respond whether the current word is same or different as in the previous 1/2/3 trial (here: 1-back). In this example the presentation of the word “passion” for the second time would require “same” response as it matches the previously presented item (n-1).. The termination of each trial depended on the response, that is participants had to respond to each word that was presented across trials and each word constituted one trial. Depending on the n-back condition, the word that was identical with the n^th^ back word required the “same” response while the other words/trials required the “different” response.

Prior to the start of the experiment (6 blocks of 128 trials each), a practice session (3 blocks of 16 trials each, data not recorded) was run. In the experiment, one of the four priming words chosen randomly and was presented for 20 milliseconds (ms) in order to prime the participants subliminally. Half of the participants were presented with secure prime word and the other half were presented with neutral prime word. In the practice session instead of presenting the prime word, a blank screen was presented for 20 ms to match the experiment protocol as the main aim was to introduce participants with the n-back task procedure. After the prime word, a mask (“XXXXX”) was presented for 500 ms to remove the visual trace of the prime word. In the beginning of each trial, the prime word was always presented prior the n-back task. After the prime word was presented subliminally and the mask was turned off, each word for the n-back task was presented for 1.000 ms. One trial in one block consisted of presenting the prime word followed by the mask and presenting words to perform the n-back task and ending with inter-trial interval before starting the next trial (see Fig 1). This trial sequence was repeated across all blocks. The task was to respond to whether the presented word is the same word or not as in the previous trial (n-1 condition), two trials before (n-2 condition), or three trials before (n-3 condition).. If the word was same, participants were asked to press the button “A” or press the button “K” if the word was different. Independent of the n-back task condition, the participants responded to each word that was presented. The response was based on whether the presented word matched the previously seen word or not. Depending on the n-back task condition (n-1/2/3), the n^th^ previously seen item required a “match” response. In other words participants had to update the WM content in order to decide whether there is a match with the current word and the n^th^ back word or not. The participants were forced to respond within 1.500 ms time window (see also Fig 1). If the response was erroneous or exceeded the time limit, error feedback was given by presenting a warning message (the word “error” in Turkish) in black for 500 ms. Each n-back task condition (n-1, n-2, n-3) was presented block-wise with attachment related and unrelated words leading to six different conditions/blocks (n-1, n-2, and n-3 tasks with attachment related and non-related words.) These six different blocks were presented in random order for each participant. As the n-back task gradually gets more difficult to perform due to the capacity of the working memory, in order to systematically increase the task difficulty, each level (1–3) of the n-back task condition was presented in the ascending order for all participants. In each n-back task condition, the order of the attachment and non-attachment related words were counterbalanced, and each word type was presented block-wise. For instance, if the first block were to be performing the n-1 task with the attachment related words, in the second block the participant would be performing the n-1 task with the non-attachment related words.

All stimuli were presented on the center of the screen in white on a gray background. Prior to the start of the next trial, 100 ms. inter-trial interval was presented. At the end of each block, participants had the opportunity to take a short break before continuing. When the experiment ended, participants filled in the ECR-R scale. The whole session lasted approximately 45 minutes.

### Design and analyses

Prior to the main analyses, we ran several ANOVAs, to check whether the gender and prime conditions affect the n-back task performance differently. To address our research questions, we used structural equation modeling (SEM) to simultaneously examine the relations among the study variables and to test the hypothesized model. Specifically, we examined if attachment dimensions predict working memory capacity across priming conditions (neutral vs. secure). First, we tested the measurement models in which reaction times and d’ values for n-1, n-2, and n-3 tasks were indicators of the processing speed and working memory capacity respectively. We computed two separate latent variables for attachment related words and non-attachment words. Second, we tested the structural model to investigate the hypotheses. Although using different working memory tasks as an indicator to define the working memory performance would be more accurate (e.g[49]), we used different task difficulty level performances as indicator of working memory capacity and processing speed in this study (see [50] for similar strategy using different task difficulty results as indicators of processing).

**Fig 2:**
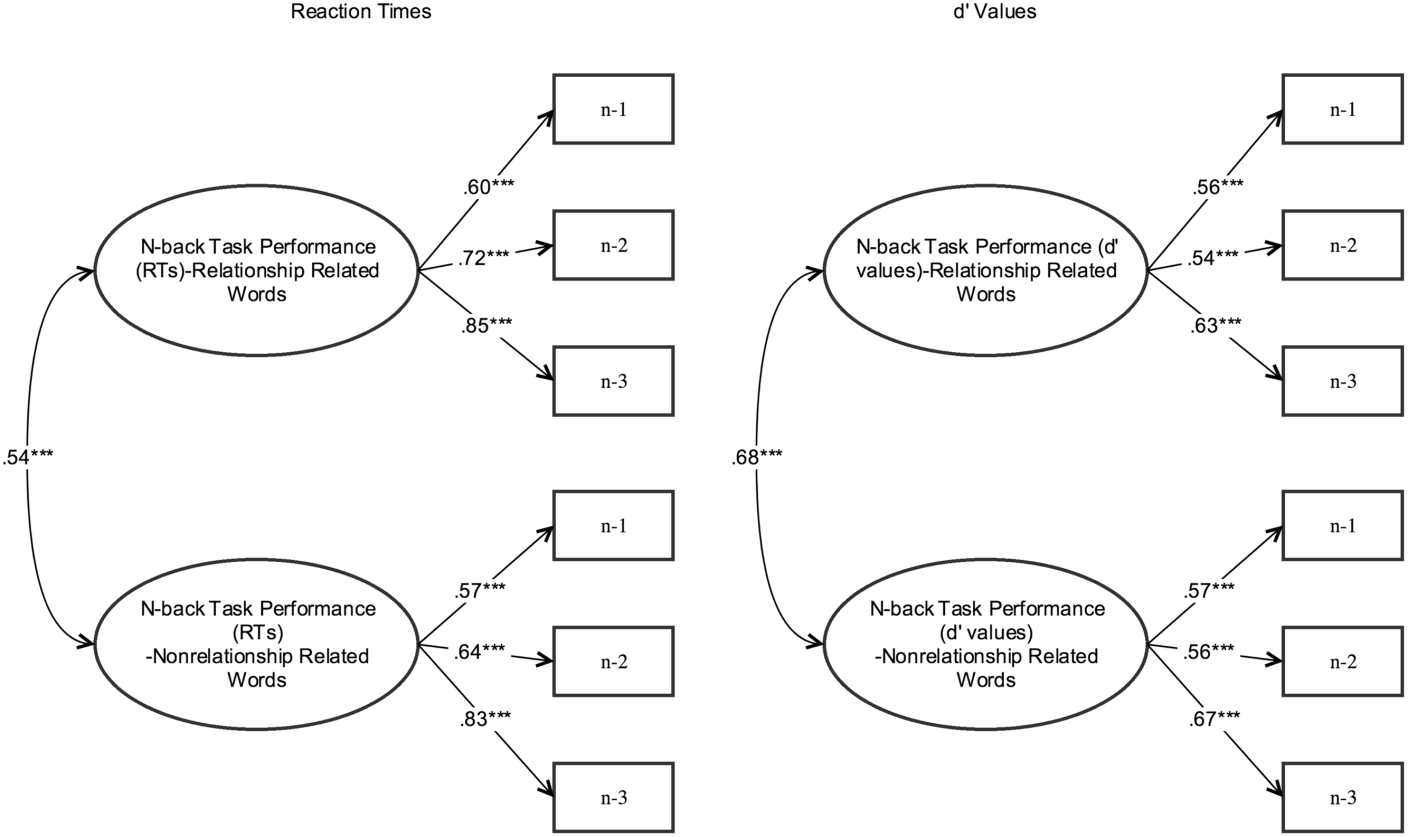
Measurement model estimations for the n-back task performance.

SEM was performed using MPlus 7.11 [51] to see if attachment dimensions (i.e., anxiety and avoidance) predict the n-back task performance (i.e., as an indicator of WM capacity). The model fit assessments were made using the Chi-Square Model Fit index, the Root Mean Square Error of Approximation (RMSEA), the Comparative Fit Index (CFI), the Tucker-Lewis index (also called as the non-normed fit index or NNFI), and the Standardized Root Mean Square Residual (SRMR). We also considered χ2 and df proportion as an additional model fit index, because the Chi-Square test is sensitive to sample size. Following previous work, RMSEA value below .06 is considered a good fit [52, 53], whereas SRMR values less than .08 are thought to be an acceptable fit [52]. Besides, the CFI is one of the most well-known fit indices, and Hu and Bentler [52] suggested that values equal to, or greater than, .90 on this index as a good fit. We utilized maximum likelihood estimation for parameters. The model was composed of two latent constructs and six observed variables (see Fig 2). Attachment dimensions, attachment anxiety and avoidance were used as observed variables in all model estimations. The indicator of the processing speed was reaction times (in ms) to n-1, n-2, and n-3 tasks. Only correct responses’ reaction times were included in the analyses. Besides, d’ values indicated sensitivity in detecting differences as reflected in accuracy in our model estimations.

## Results

Several independent sample t-tests were run to test the group differences in attachment anxiety and avoidance scores in the sample. Results showed no significant gender differences for the attachment dimensions (*t*_*anxiety*_ *(82)* = .32, *p* = .75; *t*_*avoidance*_ *(82)* = .48, *p* = .64, respectively). Secondly, two separate mixed ANOVAs were run to test if participants perform differently in different priming conditions (i.e., secure vs. neutral), gender (i.e., female vs. male), and different word types (i.e., attachment related vs. attachment non-related words). We ran these analyses for the d’ values and RTs separately. Results yielded no significant main and interaction effects for the n-back tasks in terms of the d’ values (*F*_*word type*_ *(5*,*76)* = 2.03, *p* = .08; *F*_*sex*_ *(1*,*80)* = .66, *p* = .42; *F*_*prime*_ *(1*,*80)* = .01, *p* = .94; *F*_word type*gender_*(5*,*80)* = 1.22, *p* = .31; *F*_word type*prime_*(5*,*80)* = 1.30, *p* = .27; *F*_word type*prime*gender_*(5*,*80)* = 1.75, *p* = .13), suggesting that participants’ working memory capacity did not change as a function of word type, gender and priming conditions. Similar pattern was found for the RT values, except for significant word type main effect (*F*_*word type*_ *(5*,*76)* = 11.43, *p* = .001), indicating that participants’ processing speed were faster for attachment non-related words (*M* = 526,096 ms, *SD* = 8.69) than attachment related words (*M* = 532,494 ms, *SD* = 10.25). The mean values and the standard deviations of the measured variables are shown in Table 1. Additionally, we ran two separate mixed ANOVAs to see if participants perform differently in different task difficulty conditions (i.e., n-1, n-2, n-3) and in different priming conditions (i.e., secure vs. neutral). The same analyses were run for the d’ values and RTs separately. Results yielded only significant task difficulty main effect for RTs (*F(2*,*82)* = 50.58, *p* = .000). Specifically, Bonferroni test showed that reaction time to n-3 tasks (*M* = 499.52, *SE* = 7.08) was lower than n-2 and n-1 tasks *M* = 558.27, *SE* = 6.68; *M* = 540.69, *SE* = 7.04, *respectively*).

**Table 1:** The mean values and SDs of the measured variables for the neutral (upper part) and secure priming (lower part) conditions.

***Neutral Priming***	**Attachment Related Words**			**Attachment Non-related Words**		
	**n-1**	**n-2**	**n-3**	**n-1**	**n-2**	**n-3**
**RT of hit trials (ms)**	549 (75.39)	561 (78.56)	503 (74.57)	536 (65.59)	547 (60.11)	499 (74.37)
**d’**	3.46 (1.28)	3.30 (0.86)	3.12 (0.60)	3.48 (0.80)	3.33 (1.29)	3.11 (0.69)
**Correct responses (%)**	94.04 (6.39)	93.49 (6.03)	92.80 (3.79)	94.57 (6.34)	93.29 (5.26)	92.86 (5.38)
**Errors (%)**	5.94 (6.37)	6.51 (6.03)	6.72 (3.79)	5.41 (6.35)	6.70 (5.26)	7.13 (5.38)
***Secure Priming***	**Attachment Related Words**			**Attachment Non-related Words**		
	**n-1**	**n-2**	**n-3**	**n-1**	**n-2**	**n-3**
**RT of hit trials (ms)**	555 (78.42)	567 (76.01)	500 (64.49)	533 (75.84)	557 (67.67)	496 (55.51)
**d’**	3.09 (1.67)	3.16 (1.41)	3.03 (0.58)	3.16 (1.04)	3.21 (1.39)	3.05 (0.79)
**Correct responses (%)**	89.68 (12.79)	92.04 (5.28)	92.70 (4.42)	91.82 (9.84)	92.48 (5.68)	92.32 (6.49)
**Errors (%)**	10.31 (12.79)	7.95 (5.28)	7.29 (4.42)	8.17 (9.84)	7.60 (5.73)	7.80 (6.53)

The measured variables are; reaction time (RT) in milliseconds (ms), d’ sensitivity measurement, and the percentages of correct and incorrect responses. Standard deviations are reported in brackets.

### Measurement models for processing speed and working memory capacity

As the first step in our analyses, we constructed and tested a measurement model of two latent factors (i.e., n-back task performance for attachment related words and non-attachment words in reaction times) with six measured indicator variables. Specifically, we examined if n-1, n-2, and n-3 task performances successfully represent working memory performance latent variable. We ran the same measurement model for n-back task performance in d’ values and RTs as a separate measurement model.

Measurement model with two latent factors revealed good fit for attachment related and non-attachment related word performances in reaction time base (*χ*^*2*^ (8, *N* = 84) = 11.72, p = .11; *CFI* = .98; *TLI* = .96; *RMSEA* = .09, *SRMR* = .04). Similarly, the measurement model with d’ values also yielded good fit to the data *χ*^*2*^ (8, *N* = 84) = 16.53, p = .03; *CFI* = .91; *TLI* = .89; *RMSEA* = .10, *SRMR* = .05). Overall, measurement model estimations suggested that working memory capacity latent factor could be used for the further model testing in which attachment dimensions would predict working memory capacity for attachment related and non-attachment word task performances in reaction time and d’ value bases (see Fig 2).

### Predicting working memory performance from attachment dimensions across different priming conditions

Having established the measurement models, we then tested the structural models to address our hypotheses. Specifically, we examined if attachment anxiety and avoidance would predict working memory capacity across secure and neutral priming conditions. We estimated processing speed and working memory performance on the basis of reaction time and d’ values respectively.

**Fig 3:**
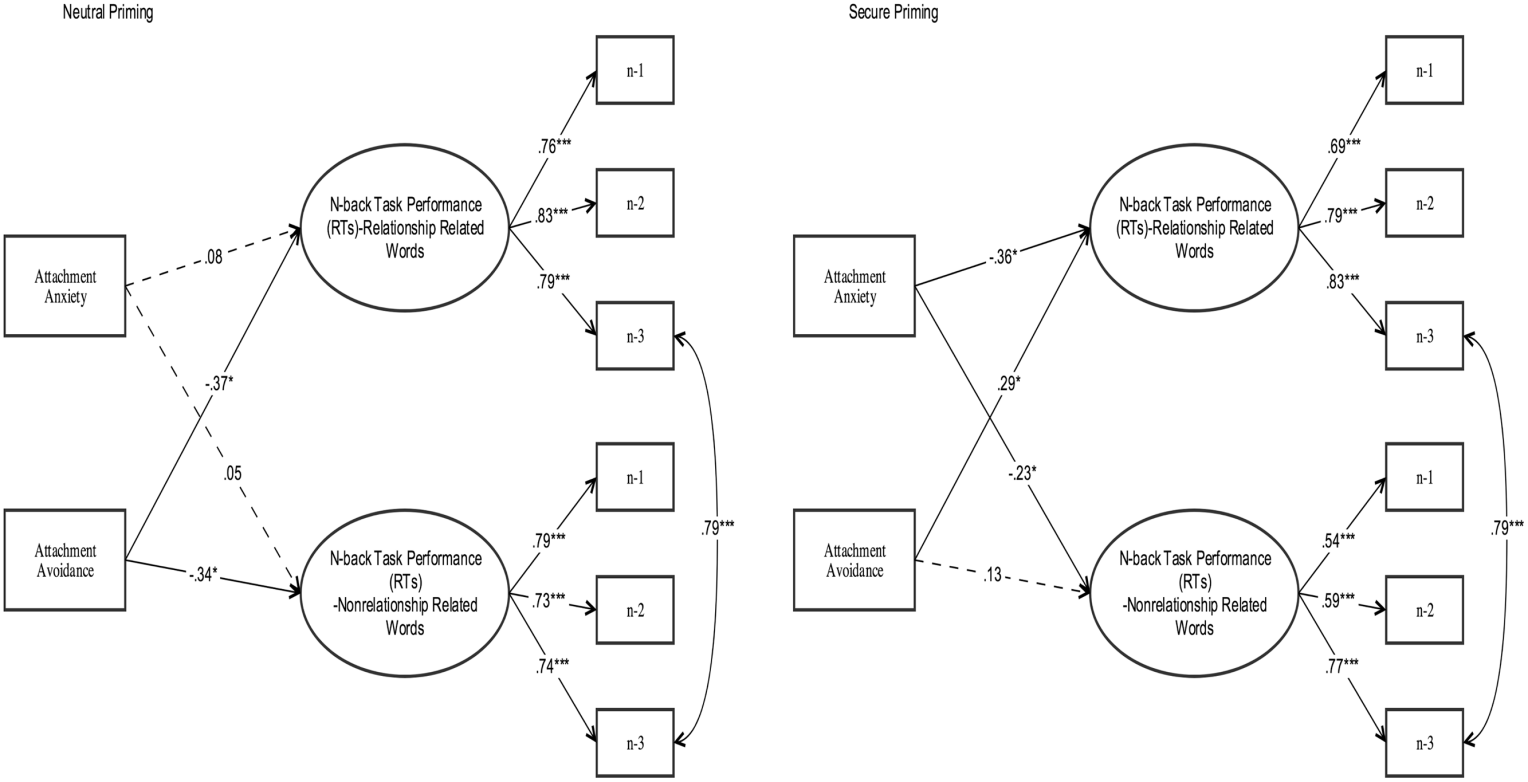
Attachment dimensions predicting the processing speed.

As depicted in Fig 3, the model yielded good fit to the data, *χ*^*2*^ (38) = 49.72, p = .10; *CFI* = .95; *TLI* = .91; *RMSEA* = .09, *SRMR* = .09. Attachment avoidance (but not anxiety) predicted n-back task performance, including attachment related (*β* = -.37, *p* < .05) and non-attachment related words (*β* = -.34, *p* < .05) in neutral priming. However, this pattern was different for the participants who were in the secure priming condition. Specifically, attachment anxiety negatively predicted reaction time in n-back task including both attachment related and non-attachment related words, indicating secure priming may foster n-back task performance of individuals who rated higher levels of attachment anxiety (see Fig 3). Besides, increase in the attachment avoidance was also associated with delay in the n-back task performance only if the task words were attachment related words in the secure priming condition (*β* = .29, *p* < .05).

**Fig 4:**
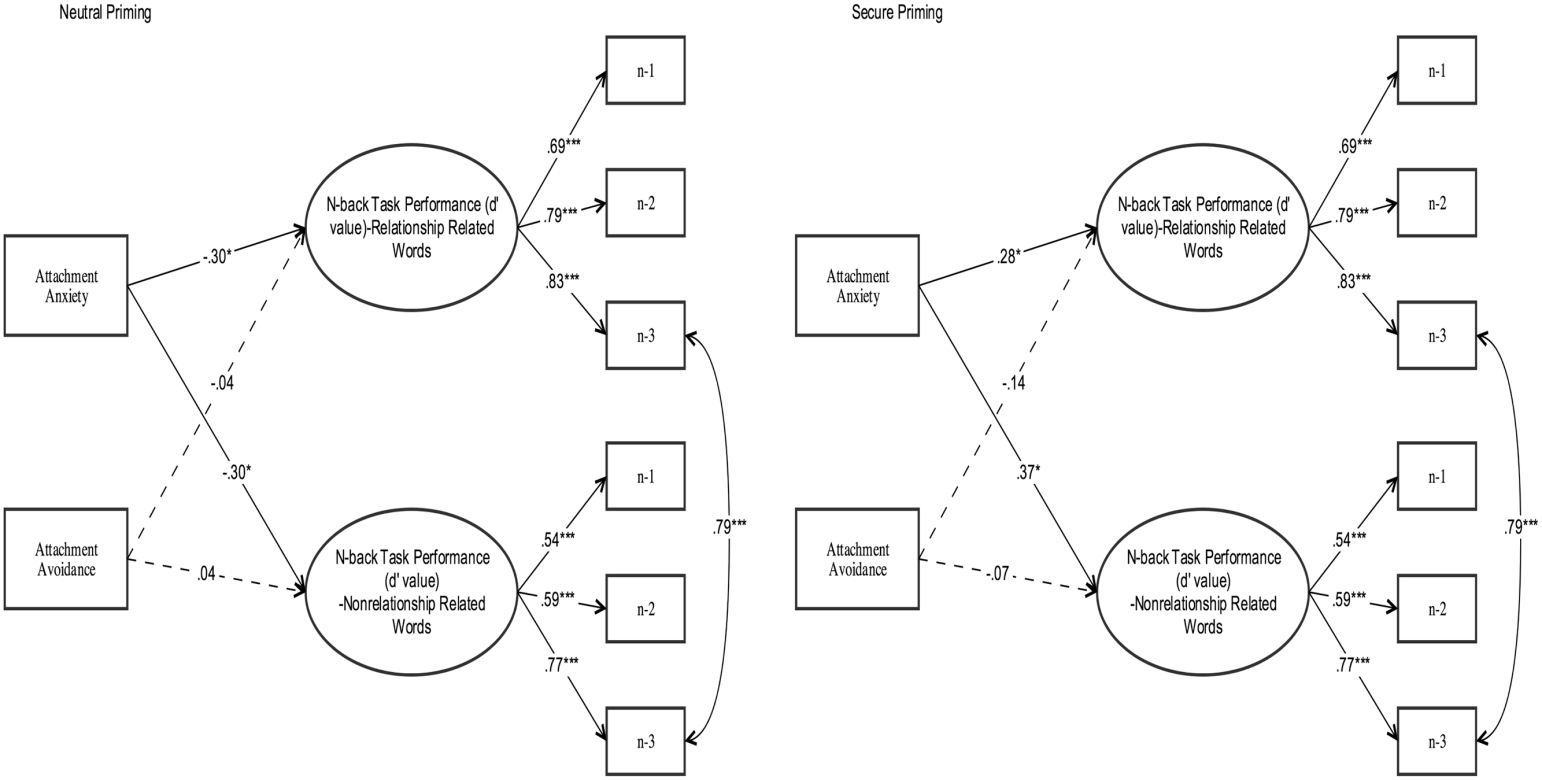
Attachment dimensions predicting the WM performance.

We also examined if attachment dimensions (i.e., anxiety and avoidance) predict n-back task performance on the basis of the d’ values. The model fit to the data adequately (*χ*^*2*^ (38) = 50.59, p = .09; *CFI* = .89; *TLI* = .88; *RMSEA* = .09, *SRMR* = .09). Specifically, attachment anxiety (but not avoidance this time) negatively predicted both attachment related (*β* = -.30, *p* < .05) and non-attachment related words (*β* = -.30, *p* < .05) n-back task performance in the neutral priming condition. In other words, in neutral priming condition, increased attachment anxiety was associated with decreased n-back task performance. In secure priming condition, this negative association between attachment anxiety and n-back task performance became reversed (see Fig 4). Specifically, after secure priming, attachment anxiety positively predicted performance in n-back tasks including both attachment related (*β* = .28, *p* < .05) and non-attachment related words (*β* = .37, *p* < .05).

## Discussion

The present study aimed to investigate whether attachment dimension of individuals (anxious vs. avoidance) would predict their working memory (WM) performance measured by the n-back task reflecting the WM capacity under either security or neutral priming conditions. The results revealed divergent patterns for different attachment dimensions (i.e., anxiety and avoidance) under the different priming conditions. Specifically, in neutral priming condition, attachment anxiety predicted poorer performance (slower RTs and lower d’ values) in the n-back task compared to secure priming condition where n-back task performance was significantly better predicted revealed by the SEM. This pattern was obtained for both attachment-related and non-related words.

Independent of the word content, being attachment related or not, attachment anxiety and avoidance showed divergent patterns in different priming conditions. In neutral priming condition, working memory performance declined in terms of capacity in the n-back task for individuals who rated higher levels of attachment anxiety. However in the secure priming condition, working memory performance was boosted in the n-back task for individuals with high attachment anxiety. These were observed in terms of the RTs and d’ values. In other words, the subliminal priming of the security led to increased WM capacity and processing speed for individuals who rated higher levels of attachment anxiety (see [24, 21] for similar results).This effect, however, was not observed in individuals with attachment avoidance. The WM performance of those individuals did not differ between neutral and secure priming conditions. This can be interpreted as a result of pre-emptive strategies, leading to the use of cognitive strategies to inhibit concepts related to attachment. Applied to the present study, increased task difficulty with relationship content did not prevent the avoidance strategies and participants did not benefit from secure priming manipulation. Similar findings were previously reported albeit without the priming manipulation. Edelstein [41] measured working memory capacity in a recall task for attachment-related, emotional and neutral words in avoidant and anxious individuals. The results revealed lower WM capacity for attachment-related words in high-avoidant, but not anxious, individuals.

The lack of benefiting from secure priming contradicts with previous findings reporting that high attachment avoidance leads to detachment from attachment related content [54, 8].. Gillath and colleagues [54] investigated inhibition and interference processes of people with different attachment orientations. Across experiments, the authors found performance benefit in individuals with attachment avoidance. Compared to anxious attachment orientation, these individuals were better at allocating attention and inhibition mechanisms. Results were interpreted as the ability of attachment avoidant individuals’ in regulating their cognitive strategies more successfully thus leading to performance benefit.

On the other hand, a recent study [10] showed that both attachment avoidance and anxiety predicted the cognitive performance negatively in the presence of threat priming in a signal detection task. Performance deterioration was observed in both anxiety and avoidance domains albeit in different ways. Attachment anxiety was associated with increased false alarm rates while attachment avoidance was associated with increased miss rates.

In the current study, higher levels of attachment avoidance predicted poorer n-back task performance for attachment-related words. This contradiction on performance benefit in certain attachment dimensions but not in others could be explained by the tasks used to measure the cognitive performance. Although a clear association exists between attachment orientation and cognitive performance, the specific cognitive task and the stimuli used affect the performance differently. The use of n-back task in the present study and the use of inhibition and interference tasks in the other study [54] and the type of the stimuli used ((non)attachment related words vs. simple letters or shapes) could account for the observed differences. The findings should be interpreted with caution, as the results may be task specific.

Previous studies showed that as the number of exposure to prime increased, the impact of it would be stronger and longer lasting [55]. Consistent with this idea, Gillath and colleagues [26] suggested that repeated priming of security may result in long-term effects and if individuals with high level of attachment anxiety could benefit from such priming. Following these results, our findings could be promising in terms of the cognitive performance of those individuals.

### Limitations and future directions

The current study was designed to investigate the relationship between attachment dimensions and cognitive performance. Working memory capacity measured by the n-back task was chosen to fulfill this goal. Although the findings provide further knowledge, the current study faces some limitations that need to be addressed in the future. Firstly, the generalizability of the findings should be tested in other domains of cognition other than working memory and also within the WM domain by using different WM tasks. Secondly, the sample of this study has limitations in two ways: it is rather a small sample and the majority consisted of female participants. Future studies should be conducted with larger sample size and with higher number of male participants to avoid any potential gender bias.

The future studies could be along the lines of investigating cognitive performance of individuals being involved in romantic relationship or not as a factor that could modify the hyperactivation and deactivation strategies.

The present study has some implications. Consistent with Fredrickson’s [56] the “broaden-and-build” cycle of attachment security, activation of attachment security via words, pictures or scenarios may enhance someone’s sense of security. By this way, repeated priming may enhance individuals’ resources for coping, cognitive flexibility, and emotional stability in times of stress [26] especially individuals who rated higher levels of attachment anxiety. Based on these discussions, our results yielded some possible protective implications of secure priming by showing that high attachment anxiety was associated with lower cognitive performance but temporary activation of security may help to improve cognitive performance and have broad implications in real-life settings.
